# Inspection and Modeling Analysis of Locking Pins in the Penultimate-Stage Blades of a 600 MW Steam Turbine

**DOI:** 10.3390/ma18194487

**Published:** 2025-09-26

**Authors:** Ke Tang, Weiwen Chen, Jiang Zhu, Binhao Yi, Qing Hao, Jiashun Gao, Zhilong Xu, Bicheng Guo, Shiqi Chen

**Affiliations:** 1 Sichuan Huadian Gongxian Power Generation Co., Ltd., Yibin 644500, China; sctk-1@163.com (K.T.);; 2 College of Marine Equipment and Mechanical Engineering, Jimei University, Xiamen 361000, China; 3 School of Marine Mechatronics, Xiamen Ocean Vocational College, Xiamen 361000, China; 4 Chengyi College, Jimei University, Xiamen 361000, China; 5 Engineering Research Center of Anti-Fatigue Manufacturing for Marine Equipment, Xiamen 361000, China

**Keywords:** steam turbine, locking pin, martensitic steel, fretting wear, stress corrosion cracking, intergranular propagation

## Abstract

The fracture behavior of a locking pin used in the penultimate-stage blades of a 600 MW steam turbine in a thermal power plant was investigated through microstructural and microhardness characterization, fracture surface and energy-dispersive spectroscopy (EDS) analysis, as well as finite element load simulation. The microhardness values measured on the cross-section of the service pins ranged from 528 to 541 HV0.1, showing little difference from the unused pins. Scanning electron microscopy analysis revealed that approximately 70% of the fracture surfaces exhibited an intergranular “rock candy” morphology. The results indicate that pin failure was primarily caused by the combined effects of fretting wear and stress corrosion cracking (SCC). Specifically, vibration at the blade root, impeller, and pins due to start–stop cycles and load variations led to fretting wear, forming pits approximately 75 μm in size. Under the combined effects of weakly corrosive wet steam environments and shear stresses, SCC initiated at the high stress concentration points of these pits. Early crack propagation primarily followed original austenite grain boundaries, while later stages mainly extended along martensite plate boundaries. As cracks advanced, the cross-sectional area gradually decreased, causing the effective shear stress to increase until it exceeded the shear strength, ultimately leading to fracture. These findings not only provide a scientific basis for enhancing the reliability of steam turbine locking pins and extending their service life, but also contribute to a broader understanding of the failure mechanisms of key components operating under corrosive and fluctuating load environments.

## 1. Introduction

In recent years, with the rapid expansion of renewable energy in the power generation sector, thermal power units, owing to their superior load regulation capacity and operational stability, have increasingly undertaken the role of deep peak regulation to accommodate fluctuating renewable energy outputs. As a result, their operating conditions have become increasingly complex and highly dynamic [1,2,3]. Frequent deep peak-regulation, rapid load fluctuations, and repeated start–stop cycles have significantly exacerbated fracture-related issues in critical turbine components [4]. Among these, the locking pin, a fundamental yet crucial mechanical fastener, is widely used across engineering structures to provide positioning, connection, and load transfer functions [5]. In steam turbines, locking pins are employed to secure blades to the disk. During service, they are subjected to multiple detrimental factors, including wet steam corrosion, centrifugal shear stress, and complex cyclic loading, which make them highly prone to fracture failure.

Constructive studies have been carried out on the failure of critical steam turbine components. For instance, one investigation on a 320 MW steam turbine revealed that blade failure was triggered by stress corrosion cracking (SCC) of the locking pin, which subsequently fractured. The fractured pin induced fretting wear, leading to blade fatigue cracking and ultimately complete blade failure [6]. Other researchers reported that blade loosening could significantly reduce its natural frequency, thereby entering a dangerous resonance range [7]. This condition promoted fretting wear at contacting blade roots, which facilitated crack initiation and propagation, eventually causing fatigue fracture of the blade root [8,9]. In another case, failure of the last-stage blades in a 660 MW steam turbine was attributed to SCC under wet steam conditions [10]. Furthermore, research on rotor reliability indicated that the design life of steam turbine rotors, when evaluated based on reliability, decreases with increasing start–stop frequency [11].

Steam turbines typically operate in high-temperature, high-humidity wet steam environments, where components are continuously exposed to weakly corrosive conditions [12,13]. To improve corrosion resistance, high-Cr martensitic steels are commonly employed for critical components [14]. Research indicates that adding chromium and other alloying elements can effectively enhance corrosion resistance, as these elements promote the formation of a dense protective oxide scale on the substrate surface. Chromium is distributed relatively uniformly within the martensitic structure, enabling the formation of a dense chromium oxide (Cr_2_O_3_) protective layer that improves corrosion resistance [15]. However, numerous studies have demonstrated that martensitic steels are highly susceptible to SCC under tensile stress in corrosive environments [16]. Further investigations revealed that SCC tends to propagate preferentially along prior-austenite grain boundaries, which often serve as the main fracture paths [17]. Microstructural and compositional analyses have additionally identified Cr-depleted zones near prior-austenite grain boundaries, which exhibit reduced corrosion resistance and act as preferential sites for SCC initiation and growth [18,19]. Thus, the characteristics of prior-austenite grain boundaries and their surrounding compositional distribution are key factors governing the SCC resistance of martensitic steels.

**Figure 1:**
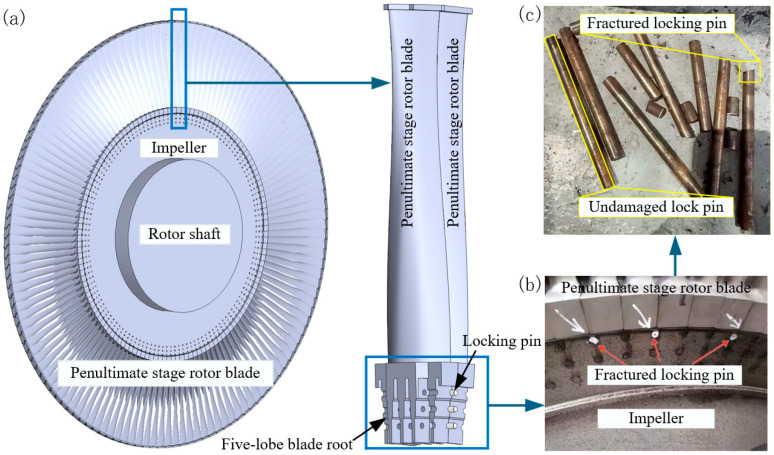
Locking pin and its assembly relationship with rotor blades and the wheel: (**a**) schematic diagram of the assembly; (**b**) photograph of the assembled components; (**c**) fractured locking pin.

During the overhaul of a 600 MW thermal power unit, large-scale fractures were observed in the locking pins of the penultimate-stage five-fork rotor blades after nine years of service. Among the 798 pins at this stage, 61 were broken. The failure mainly occurred in the outer bifurcation area of the five-pronged blade root, posing a serious threat to the unit operation (Figure 1). To identify the root cause of the pin failures, this study focuses on the failed H11 steel pins. A comprehensive investigation was carried out, including hardness testing, microstructural characterization, fracture surface analysis, energy-dispersive spectroscopy (EDS), and load simulation analysis, to elucidate their degradation behavior and failure mechanisms in the wet steam environment. The findings of this work provide a basis for enhancing the service reliability of H11 steel pins.

## 2. Materials and Methods

### 2.1. Materials

The locking pins were made of H11 steel, in accordance with ASTM A681-08 (2022) [20]. The quenching treatment was performed at 1050 °C with a holding time of 1 h, followed by oil cooling. The tempering treatment was carried out at 550 °C with a holding time of 2.5 h, followed by air cooling. Two types of samples were selected: (i) fractured locking pins that failed during service, and (ii) non-fractured locking pins used as controls. The chemical composition of the pins was analyzed using an optical emission spectrometer (SpectroMAXx BT, SPECTRO Analytical Instruments GmbH, Kleve, Germany). The chemical composition of the locking pin material is listed in Table 1. The diameter of the pin is 10.35 mm and its length is 146 mm.

### 2.2. Methods

**Figure 2:**
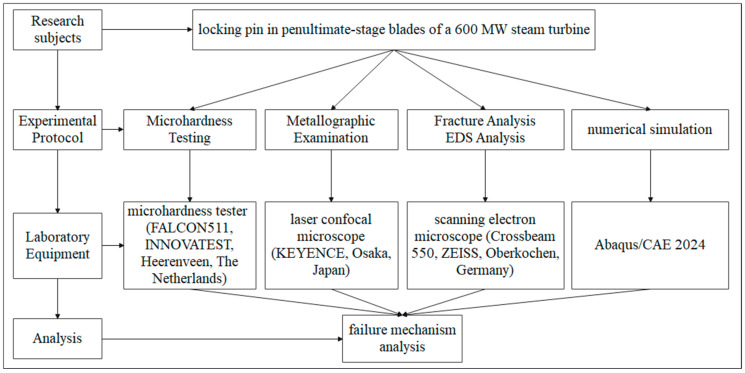
Research Flowchart.

A series of characterization and testing methods were employed to study the locking pins, including hardness measurement, microstructure analysis, fracture morphology observation, EDS elemental analysis, and finite element load simulation, as shown in Figure 2.

First, Vickers microhardness testing was performed using a microhardness tester (FALCON511, INNOVATEST, Heerenveen, The Netherlands) under a load of 100 gf with a dwell time of 15 s. Measurements were conducted at the central regions of the cross-sections from the front, middle, and rear segments of both fractured and non-fractured locking pins. For each sample, three points were tested and averaged to evaluate the effectiveness of heat treatment and service-induced hardness variations.

Second, metallographic samples were prepared from the same cross-sectional regions of fractured and non-fractured pins. The specimens were mounted, ground, and polished, followed by etching with a stainless-steel etchant (composition: 60% water, 30% hydrochloric acid, and 10% ferric chloride) for 10 s, then rinsed with alcohol and air-dried. The microstructural features, including tempered lath martensite, grain morphology, inclusions, and other defects, were examined using a laser confocal microscope (VK-X1000, KEYENCE, Osaka, Japan).

Fractographic analysis of fractured locking pins was carried out using a scanning electron microscope (SEM) (Crossbeam 550, ZEISS, Oberkochen, Germany). The fracture surfaces were divided into crack initiation, crack propagation, and final rapid fracture regions. EDS was performed in selected areas to identify elemental distributions and determine whether specific elements promoted crack growth.

All metallographic, hardness, and fracture surface analyses were conducted under laboratory conditions at room temperature (24 ± 2 °C) and relative humidity of 40–60%. The EDS analyses were carried out in a high-vacuum environment to ensure measurement accuracy.

**Figure 3:**
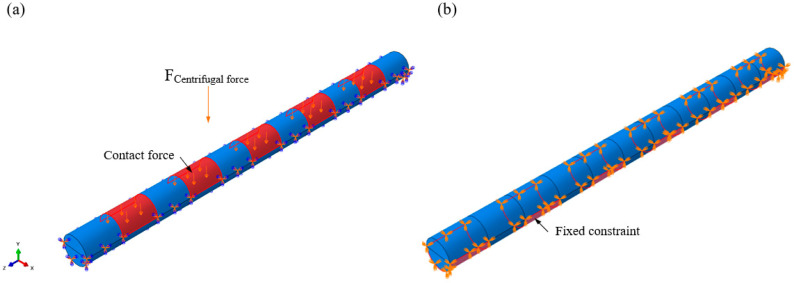
Load and boundary conditions of the finite element model: (**a**) Locking pin subjected to centrifugal forces transmitted from the blade. (**b**) Fixed boundary conditions applied at the wheel–pin contact regions.

Furthermore, finite element analysis (FEA) of the locking pin was conducted using Abaqus/CAE 2024 software. In the model, the rotor was assumed to be rigidly fixed, while the blade tip was subjected to centrifugal forces. The locking pin was connected to the rotor through an interference fit, and centrifugal loading from high-speed blade rotation was applied to the pin. The stress distribution within the locking pin was analyzed to identify high stress concentration regions, thereby providing mechanical evidence for the observed failure locations. The load and boundary conditions of the simulation model are shown in Figure 3. The locking pin is subjected to the centrifugal force transmitted from the blade root, while the regions in contact with the wheel are fixed to represent the actual assembly constraints. A hexahedral mesh was used to establish mesh cells of different sizes for comparison, namely 0.6 mm, 0.8 mm, and 1 mm resulting in 63,404, 29,546 and 15,158 elements, respectively. The material parameters were set as follows: Young’s modulus of 205,000 MPa and Poisson’s ratio of 0.3. In the simulation, the pin–rotor interface was constrained as fixed, while a normal surface load of 100 MPa was applied to the pin–blade interface to simulate centrifugal loading during blade operation. The simulations were executed on a Windows 11 (64-bit) workstation equipped with an NVIDIA GeForce RTX 4060 and 32 GB RAM.

## 3. Results

### 3.1. Chemical Composition and Microhardness

**Figure 4:**
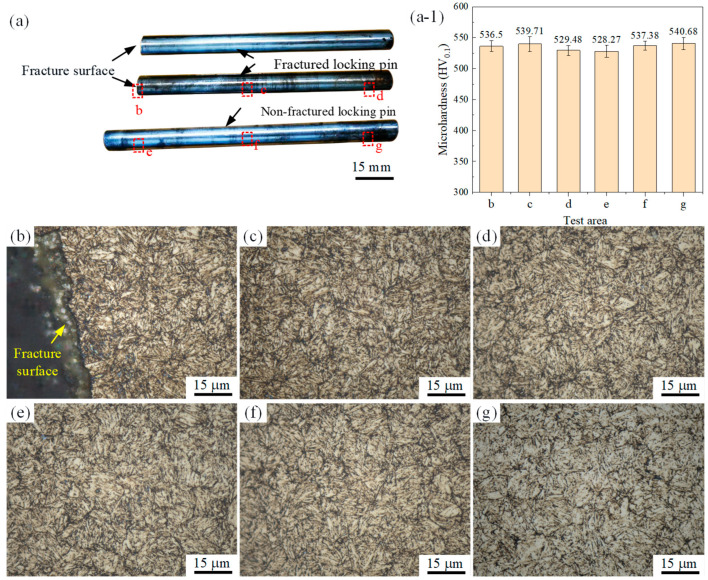
(**a**) Fractured lock pin and unfractured lock pin; (**a-1**) Hardness Comparison Between Fractured and Unfractured Lock Pins in Different Regions; (**b**–**g**) Microstructural features of fractured and non-fractured locking pins.

The chemical analysis results indicate that the materials of both the fractured and non-fractured locking pins meet the requirements of H11 steel specified in ASTM A681-08 (2022), as listed in Table 1. For microhardness testing, three regions were selected on both the fractured and non-fractured pins, as marked by the red dashed boxes in Figure 4a. The measured microhardness values ranged from 528 to 541 HV0.1 with only minor fluctuations, suggesting a relatively uniform hardness distribution across the material, as shown in Figure 4a-1. Notably, the microhardness near the fracture region (region b in Figure 4a-1) was 536.5 HV0.1, which is essentially consistent with the other regions, indicating that no temper softening or hardness anomaly occurred in the fracture zone [6].

### 3.2. Microstructural Analysis

The microstructure within the red dashed region in Figure 4a was examined, and the results are presented in Figure 4b–g. Both groups of locking pins exhibit a characteristic tempered martensitic structure, which is compact and homogeneous, without evidence of decarburization, grain coarsening, or carbide precipitation. These observations confirm that the heat treatment process was properly executed and free from technological defects. Furthermore, the microstructure adjacent to the fracture surface shows no significant difference from that in regions farther away, and no indications of abnormal grain growth, overheating, or coarse carbide segregation were detected. Therefore, it can be inferred that the fracture was not attributable to microstructural anomalies, as illustrated in Figure 4b.

### 3.3. Fracture Morphology

**Figure 5:**
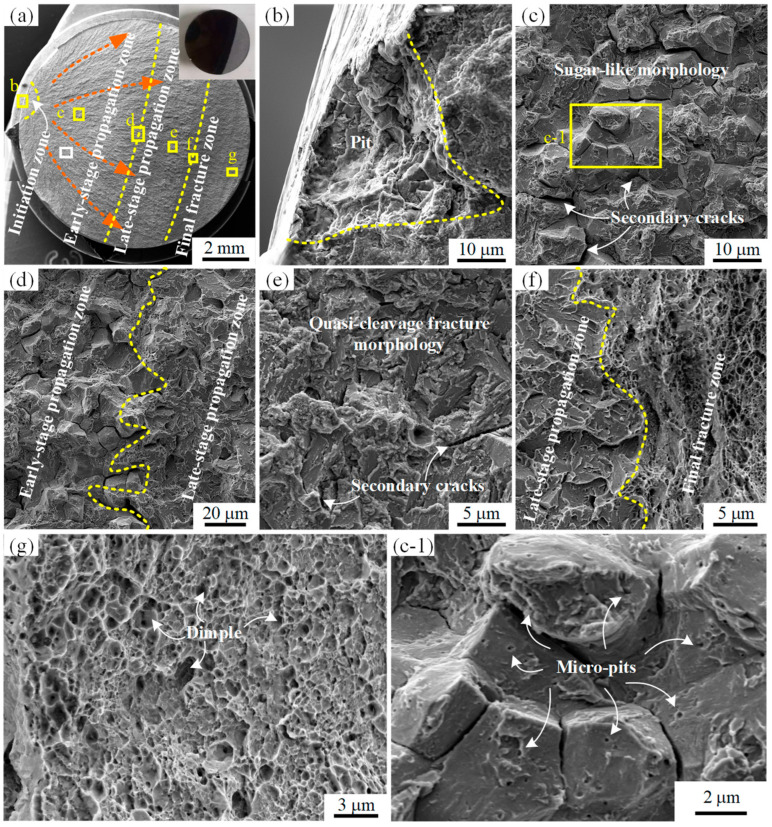
Fracture morphology of the fractured locking pin: (**a**) Overall fracture surface, with red arrows indicating radial markings of crack propagation; the inset in the upper right corner shows the visual inspection result. (**b**–**g**) Enlarged views of the yellow-boxed region in (**a**). (**c-1**) Further magnified view of the yellow-boxed region in (**c**).

Visual inspection of the fractured locking pin revealed that the fracture surface consisted of a black region corresponding to an old fracture and a gray region corresponding to a new fracture, as shown in the upper right corner of Figure 5a. Further SEM fractographic analysis indicated that the entire fracture surface could be divided into four distinct regions: crack initiation zone, early-stage propagation zone, late-stage propagation zone, and final fracture zone. Clear radial markings emanating from the crack initiation zone were observed, as indicated by the red arrows in Figure 5a. A trumpet-shaped pit was identified at the crack initiation site, with an opening diameter of approximately 75 μm and a depth of ~60 μm, as shown in Figure 5b. The early-stage propagation zone exhibited a typical intergranular fracture morphology with rock-sugar-like features, along with multiple secondary cracks, as presented in Figure 5c [21]. A distinct boundary without any transition region was observed between the early- and late-stage propagation zones, as shown in Figure 5d. The late-stage propagation zone displayed a pronounced quasi-cleavage fracture morphology with the presence of numerous secondary cracks, as illustrated in Figure 5e. A clear boundary without transition was also observed between the late-stage propagation zone and the final fracture zone, as shown in Figure 5f. The final fracture zone exhibited ductile transgranular fracture features with well-developed dimples, as shown in Figure 5g. Moreover, the fracture surface of the early-stage propagation zone was densely covered with uniformly distributed nanopits smaller than 200 nm in diameter, as shown in Figure 5c-1.

**Figure 6:**
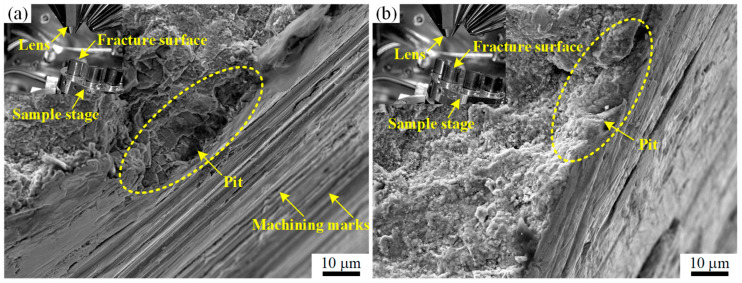
Fracture initiation zone morphology of the fractured locking pins: (**a**) fracture initiation zone of one pin; (**b**) fracture initiation zone of another pin. The insets in the upper-left corners of both figures illustrate the relative position between the SEM lens and the specimen stage.

To analyze whether the crack initiated from pits, the fracture initiation zones of several pins were examined. By adjusting the tilt angle of the SEM stage, both the fracture morphology and the outer surface of the pin could be captured simultaneously, as shown in the upper-left insets of Figure 6a,b. The results revealed that pits existed in the crack initiation zones of two pins, as shown in Figure 6a,b. Moreover, machining marks were observed on the outer surface of the pins, and the pits interrupted the otherwise continuous machining marks, as shown in Figure 6a.

### 3.4. EDS Analysis

**Figure 7:**
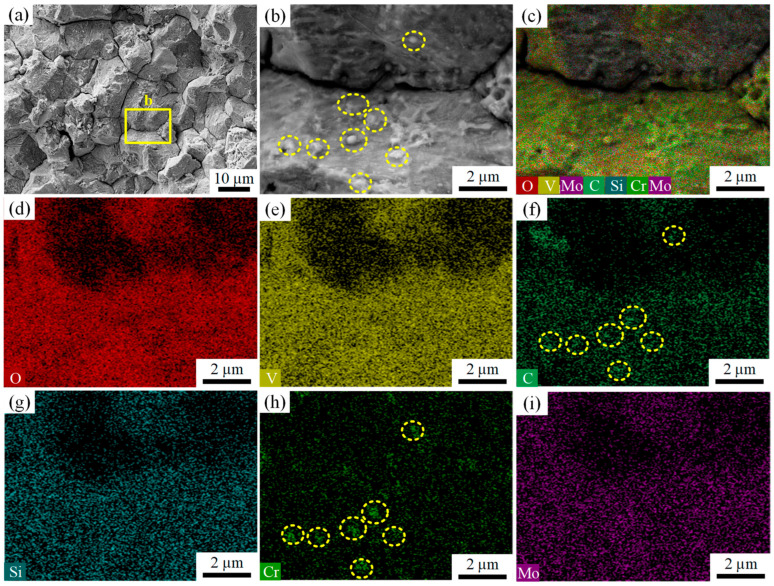
Morphology of the early-stage crack propagation zone and EDS analysis of the fractured locking pin: (**a**) rock-candy-like intergranular fracture morphology; (**b**) nanoparticles observed on the fracture surface; (**c**–**i**) elemental distribution maps from EDS analysis of region (**b**).

**Figure 8:**
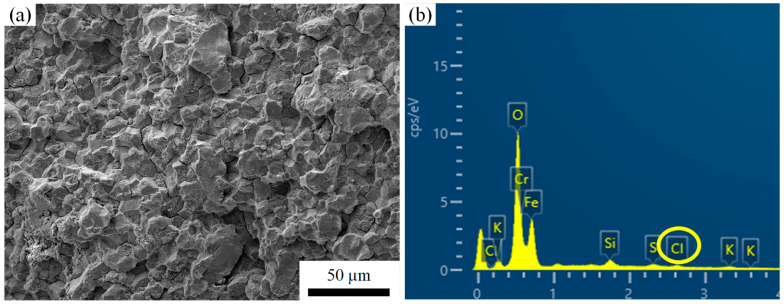
Morphology and EDS analysis of the early-stage crack propagation zone of the fractured locking pin: (**a**) rock-candy-like intergranular fracture morphology; (**b**) EDS results of the overall region shown in (**a**).

The white rectangular region in Figure 5a was further magnified, where pronounced sugar-like intergranular fracture features and secondary cracks were again observed, as shown in Figure 7a. Further magnification of the yellow rectangular region in Figure 7a revealed a large number of nanoscale particles with diameters smaller than 200 nm distributed on the fracture surface, as illustrated in Figure 7b. EDS analyses were conducted on the fracture surface shown in Figure 7b, and the corresponding results are presented in Figure 7c–i. As indicated by the EDS results, the nanoscale particles on the fracture surface were enriched in Cr and C compared with the surrounding regions. In addition to Cr, Si, Mo, V and C, a significant amount of O was also detected within the analyzed region, as shown in Figure 7d. In the EDS analysis of the crack propagation zone shown in Figure 8a, the presence of Cl was identified in addition to O, as illustrated in Figure 8b.

### 3.5. Load Analysis

**Figure 9:**
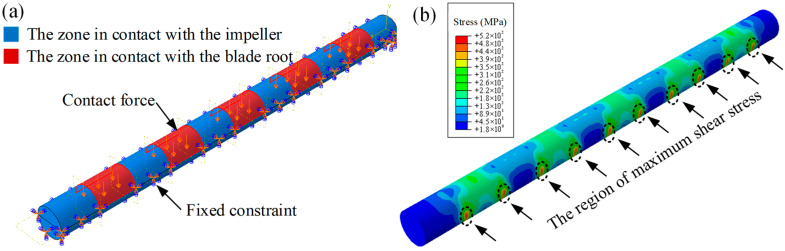
Load analysis of the locking pin: (**a**) applied loading and boundary constraints; (**b**) FE simulation results. The black dashed regions indicate the locations of maximum shear stress (mesh: 29,546 elements, 33,652 nodes).

To verify whether the locking pin experienced any structural loading anomalies under normal operating conditions, a simplified finite element (FE) model of the turbine blade locking pin assembly was established, as shown in Figure 9. The modeling, meshing, and load analysis were performed using Abaqus, with a focus on evaluating the stress distribution of the locking pin under centrifugal loading from the blade. The 3D model of the locking pin was discretized into 29,546 hexahedral elements and 33,652 nodes.

Since the root of the penultimate-stage rotor blade adopts a five-lug configuration, the locking pin was divided into 11 segments: five alternating segments were in contact with the blade root, while the remaining six segments interfaced with the disk. For boundary conditions, the disk-contacting regions were subjected to fully fixed constraints, while surface loads were applied to the blade-root-contacting regions to equivalently simulate the centrifugal force exerted on the locking pin during rotation, as shown in Figure 9a.

The simulation results indicated that the equivalent stress distribution along the pin was generally uniform and exhibited a periodic pattern. The maximum stress was concentrated at the junctions between adjacent segments, corresponding to the regions subjected to shear stress from both the blade root and the disk, as illustrated in Figure 9b. 

**Figure 10:**
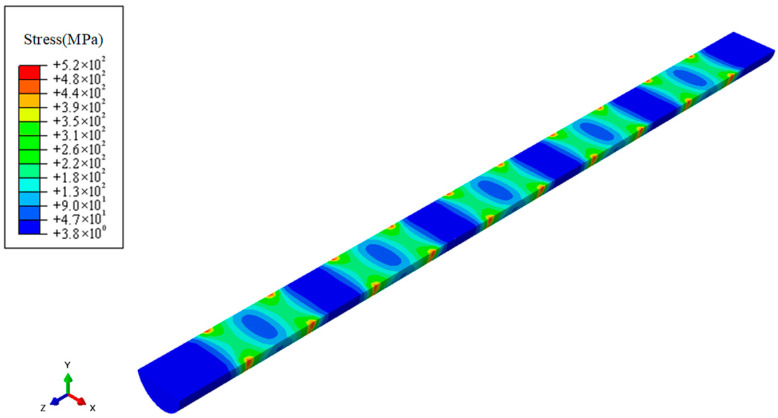
Shear Stress Distribution.

Additionally, to more intuitively illustrate the stress transfer characteristics within the locking pin, this study performed planar sectioning on the model, with the results shown in Figure 10. The stress distribution cross-section clearly reveals that the internal stress concentration zones align with the overall stress distribution trend, further validating the model’s predictive accuracy.

**Figure 11:**
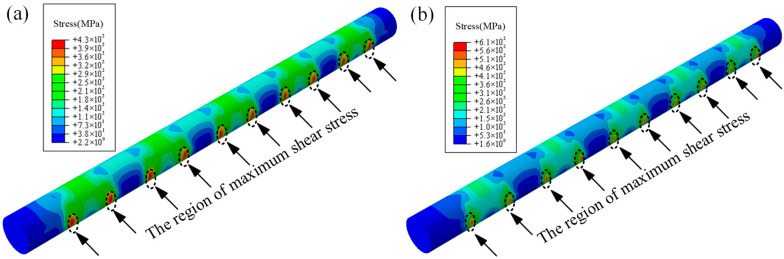
Results of mesh independence verification: (**a**) mesh size with 15,158 elements and 17,856 nodes; (**b**) mesh size with 63,404 elements and 70,470 nodes.

To eliminate the influence of mesh size on the analysis results, a mesh independence study was conducted by generating different mesh densities, while keeping all other parameters constant. The results demonstrated that the location of maximum stress remained unchanged across different mesh sizes, as shown in Figure 11.

## 4. Discussion

### 4.1. Formation of Pits

The crack initiation of the fractured locking pin was observed in the pitted region, which was located approximately 10–15 mm from the exhaust side end of the pin. This region overlaps with the interface between the blade root and the impeller. Frequent deep peak-load regulation and start–stop operations of the turbine, in response to grid demands, induced blade vibrations. These vibrations promoted fretting wear at the blade root–disk interface, eventually leading to pit formation [22].

**Figure 12:**
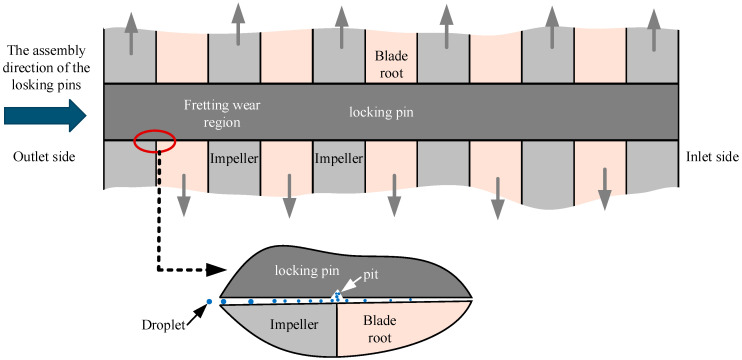
Pit formation induced by the synergistic effect of fretting wear and corrosion.

The fit between the pin and the hole was a transitional fit, completed on site during installation. Specifically, the pin diameter was first measured, then the hole was reamed based on the transitional fit requirements, and finally the pin was driven into the hole from the steam outlet side. This installation method caused the hole diameter on the steam outlet side to be larger than that on the steam inlet side, thereby creating a clearance in the hole–shaft fit on the exhaust side. Such clearance made the steam outlet side more susceptible to fretting wear and pit formation, as shown in Figure 12. The pits observed in Figure 6 further confirm this phenomenon.

Moreover, the clearance in the hole–shaft fit facilitated the ingress of steam into the region. The Cl^−^ ions present in the steam promoted corrosion of the pin material, as evidenced by the EDS analysis in Figure 8b. The combined action of fretting wear and corrosion not only caused mechanical damage but also disrupted the protective oxide film on the metal surface, thereby accelerating pit formation [23,24].

### 4.2. Crack Initiation and Propagation

Cracks were initiated in the pit region, where the stress concentration factor was high, as confirmed by the fracture analysis in Figure 5a,b. The pit region coincided with the maximum shear stress zone, as evidenced by Figure 9 and Figure 11. Under the combined action of shear stress and steam corrosion, SCC was readily triggered [25,26,27,28]. Since SCC is highly sensitive to stress concentration, cracks nucleated at the pit tips under the action of SCC [29].

Once nucleated, cracks propagated rapidly in the early stage along the prior-austenite grain boundaries under the influence of SCC, as shown in Figure 5c. With further crack extension, the effective load-bearing cross-sectional area of the locking pin decreased, leading to increased shear stress. Consequently, the crack propagation mode transitioned from intergranular propagation along prior-austenite grain boundaries in the early stage to propagation primarily along martensitic lath boundaries in the later stage, as supported by the fracture morphologies in Figure 5d,e. With continued crack growth, the actual stress eventually exceeded the shear strength, resulting in final plastic fracture, as evidenced by the dimple features in Figure 5f,g.

**Figure 13:**
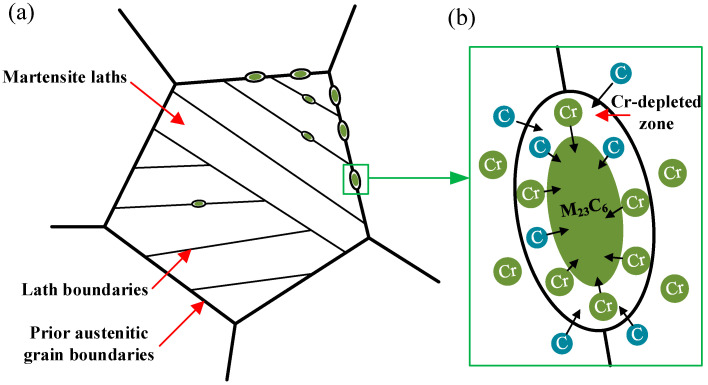
(**a**) M23C6 carbides precipitate along the original austenite grain boundaries and martensite plate boundaries; (**b**) Formation of Cr-depleted zones.

The study of stress corrosion crack propagation by Venezuela et al. [30] shows that the cracks are usually along the original austenite grain boundaries and martensite. The slat boundary expands rapidly. For SCC, inappropriate heat treatment processes may induce the formation of chromium-depleted (Cr-depleted) zones, thereby increasing the SCC susceptibility of the material [31]. Specifically, tempering within the sensitization temperature range can lead to the precipitation of M23C6-type Cr carbides along prior-austenite grain boundaries and martensitic lath boundaries [32], as shown in Figure 13a. During this process, the diffusion of C and Cr atoms toward the boundaries promotes carbide formation. However, since Cr diffuses more slowly than C, the surrounding regions experience significant Cr depletion that cannot be replenished in time, thus forming Cr-depleted zones, as illustrated in Figure 13b.

**Figure 14:**
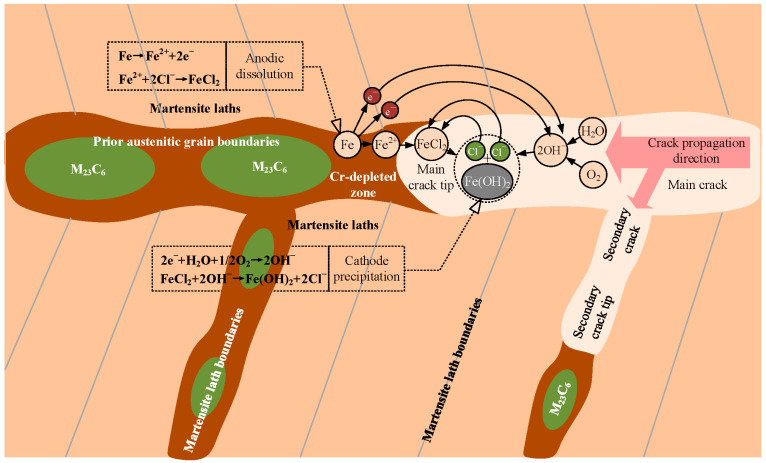
Corrosion along prior-austenite grain boundaries and martensite lath boundaries.

In corrosive environments, Cr-depleted zones act as anodic sites that preferentially dissolve, while other regions act as cathodes, resulting in galvanic effects [33,34]. Because these Cr-depleted zones are primarily distributed along prior-austenite grain boundaries and martensitic lath boundaries, the risk of localized corrosion along these boundaries increases, as shown in Figure 14.

**Figure 15:**
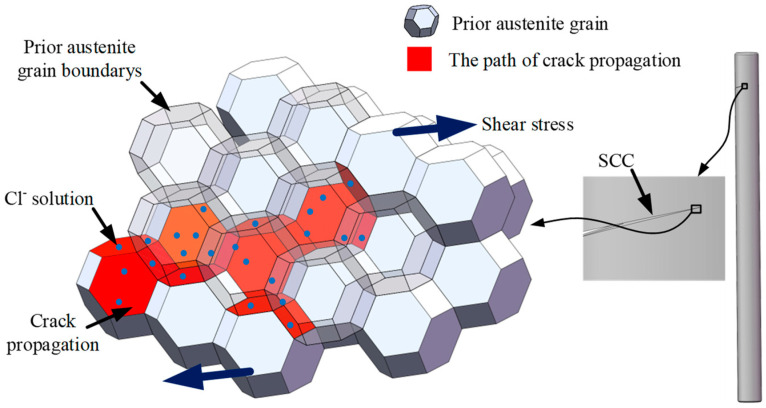
Crack propagation along prior-austenite grain boundaries under stress corrosion.

Given the high-moisture service environment of the locking pins, the clearance at the steam outlet side makes this region more prone to contact with steam. The presence of Cl^−^ ions in the steam promotes corrosion of the Cr-depleted zones, rendering the prior-austenite grain boundaries weak points for corrosion resistance. Under shear stress, the stress concentration at pit tips further increases their susceptibility to SCC, leading to intergranular crack propagation, as shown in Figure 15.

## 5. Conclusions

This study investigated the fracture behavior of H11 steel locking pins used in turbine blades under service conditions, using hardness testing, microstructural analysis, fracture surface characterization, EDS analysis, and finite element load simulations. The following conclusions were drawn:

1. The fracture of the locking pins was primarily caused by fretting wear and stress corrosion. Specifically, vibrations during deep load-following operations and start–stop cycles led to fretting wear at the junction of the blade root, impeller, and pin, forming pits.
2. Under the coupled action of a moist steam environment and shear stress, SCC was initiated at the highly stressed pit tips, and the cracks initially propagated along prior-austenite grain boundaries.
3. As cracks extended further, the effective load-bearing cross-sectional area of the pin decreased, leading to an increase in shear stress. Consequently, crack propagation transitioned from primarily intergranular growth along prior-austenite grain boundaries to propagation along martensitic lath boundaries.
4. With continued crack growth, the unfractured cross-section diminished, the actual shear stress increased, and once it exceeded the shear strength, final plastic fracture occurred.
5. This study deepens the understanding of fatigue and fracture mechanisms in high-chromium martensitic steel under service conditions. This work contributes to enhancing the safety and reliability of steam turbines while also providing a theoretical basis for strengthening locking pins.

## 6. Suggestions

1. Improve the shear strength and corrosion resistance of the locking pin material to enhance resistance against shear-induced SCC. For example, high-frequency induction quenching followed by tempering could be applied to increase both shear strength and corrosion resistance.
2. Strictly control the Cl^−^ concentration and oxygen content in the water source to reduce the corrosion rate, thereby mitigating the progression of SCC.

## Figures and Tables

**Table 1:** Chemical composition of the locking pin material (wt.%).

Content	C	Si	Mn	P	Cr	Ni	V	Mo
Specified value	0.33~0.43	0.80~1.25	0.20~0.50	≤0.030	4.75~5.50	≤0.25	0.30~0.60	1.10~1.60
Fractured locking pin	0.41	0.95	0.396	0.008	4.95	0.21	0.55	1.1
Non-fractured locking pin	0.39	0.97	0.410	0.009	5.01	0.19	0.46	1.3

## Data Availability

The original contributions presented in this study are included in the article. Further inquiries can be directed to the corresponding author.
